# The Uyghur population and genetic susceptibility to type 2 diabetes: potential role for variants in *CAPN10*,*APM1* and *FUT6* genes

**DOI:** 10.1111/jcmm.12911

**Published:** 2016-07-04

**Authors:** Feifei Zhao, Dolikun Mamatyusupu, Youxin Wang, Honghong Fang, Hao Wang, Qing Gao, Hao Dong, Siqi Ge, Xinwei Yu, Jie Zhang, Lijuan Wu, Manshu Song, Wei Wang

**Affiliations:** ^1^ School of Public HealthCapital Medical UniversityBeijingChina; ^2^ Municipal Key Laboratory of Clinical EpidemiologyBeijingChina; ^3^ College of the Life Sciences and TechnologyXinjiang UniversityUrumqiChina; ^4^ School of Medical SciencesEdith Cowan UniversityJoondalupWAAustralia

**Keywords:** type 2 diabetes, susceptibility loci, *CAPN10*, *APM1*, *FUT6*, Uyghur

## Abstract

Genome‐wide association studies have successfully identified over 70 loci associated with the risk of type 2 diabetes mellitus (T2DM) in multiple populations of European ancestry. However, the risk attributable to an individual variant is modest and does not yet provide convincing evidence for clinical utility. Association between these established genetic variants and T2DM in general populations is hitherto understudied in the isolated populations, such as the Uyghurs, resident in *Hetian,* far southern *Xinjiang Uyghur Autonomous Region*, China. In this case–control study, we genotyped 13 single‐nucleotide polymorphisms (SNPs) at 10 genes associated with diabetes in 130 cases with T2DM and 135 healthy controls of Uyghur, a Chinese minority ethnic group. Three of the 13 SNPs demonstrated significant association with T2DM in the Uyghur population. There were significant differences between the T2DM patients and controls in the risk allele distributions of rs3792267 (*CAPN10*) (*P* = 0.002), rs1501299 (*APM1*) (*P* = 0.017), and rs3760776 (*FUT6*) (*P* = 0.031). Allelic carriers of rs3792267‐A, rs1501299‐T, and rs3760776‐T had a 2.24‐fold [OR (95% CI): 1.35–3.71], 0.59‐fold [OR (95% CI): 0.39–0.91], 0.57‐fold [OR (95% CI): 0.34–0.95] increased risk for T2DM respectively. We further confirmed that the cumulative risk allelic scores calculated from the 13 susceptibility loci for T2DM differed significantly between the T2DM patients and controls (*P* = 0.001), and the effect of obesity/overweight on T2DM was only observed in the subjects with a combined risk allelic score under a value of 17. This study observed that the SNPs rs3792267 in *CAPN10*, rs1501299 in *APM1*, and rs3760776 in *FUT6* might serve as potential susceptible biomarkers for T2DM in Uyghurs. The cumulative risk allelic scores of multiple loci with modest individual effects are also significant risk factors in Uyghurs for T2DM, particularly among non‐obese individuals. This is the first investigation having observed/found genetic variations on genetic loci functionally linked with glycosylation associated with the risk of T2DM in a Uyghur population.

## Introduction

As a common heterogeneous disease, type 2 diabetes mellitus (T2DM) has become a global health catastrophe threatening economies especially in those of low‐ and middle‐income countries in which more than 80% of diabetes deaths occur 1. It is estimated that the number of people with diabetes worldwide will rise to 552 million in the year of 2030 if no urgent action is taken, and meanwhile diabetes will be the 7th leading cause of death 2, 3. In China, 9.7% of Chinese adults are suffering from T2DM, 60.7% of them are unaware, and 15.5% have pre‐diabetes at risk of cardiovascular diseases 4. T2DM is characterized by two fundamental features: insulin resistance and progressive pancreatic β‐cell dysfunction, corresponding defects in both insulin action and secretion respectively 5. For the time being, ample evidence suggests that T2DM origins with an interaction between genetic (*i.e*., family history of diabetes) and environmental determinants (*i.e*., low physical exercise, smoking, high fat intake). Subsequent association studies on the role of genetic variants to predict T2DM in certain ethnic groups have produced conflicting results 6, 7, 8, 9, 10. Thereby, it is imperative to explore and define population‐specific genetic and environmental risk factors, which will shed light on the pathogenesis of T2DM.

Recently, the research of glycomics is becoming an important focus in different fields of biology and medicine. *N*‐linked oligosaccharides of glycoproteins (*N*‐glycans) based on individual background variability and inherent sensitivity reflect the integrative effect of both genetic and environmental factors on the individuals, so as to make *N*‐glycans promising disease biomarkers 11, 12, 13, 14. Italian National Research Center has proved the application of serum *N*‐glycan profiles, especially those of fucose‐containing glycans, as sensitive surrogate biomarker for the presence of diabetes and metabolic syndrome 15. Currently, except one report on *N*‐Glycan profiling of metabolic syndrome in Chinese Han population 16, there is no study to link *N*‐glycan profiles with these factors with diabetes from Chinese ethnic minorities. Thereby, there might be differences in the contribution of known single‐nucleotide polymorphisms (SNPs) associated with fucosylation among various ethnic populations 17, 18.

In history, *Xinjiang*, in the northwestern frontier area of China, served as the key controlling section of the well‐known Silk Road, which was an ancient network of trade and cultural transmission routes that were central to cultural interaction through regions of the Asian continent connecting the West and East by merchants, pilgrims, monks, soldiers, nomads, and urban dwellers from China and India to the Mediterranean Sea during various periods of time 19. Consequently, the ethnic minorities including Uyghurs in *Xinjiang* became admixed populations with both eastern and western Eurasian ancestries 20. However, as time goes on, the Uyghurs, living in *Hetian*, far southern *Xinjiang Uyghur Autonomous Region*, have been practicing consanguinity and endogamy since they settled down in the area some 2000–2500 years (80–100 generations) ago, and are highly distinguishable from both current Europeans and East Asians due to the endogamy and genetic isolation in terms of the time scale of historical events 20, 21. Therefore, the Uyghurs are a classically well‐defined isolated population, practicing endogamy resident in a relatively homogeneous environment and have large sib ships. And they are overwhelmingly Muslim, and have their own language, religious beliefs, and lifestyles that are very different from either Han Chinese population or American/European populations 18, 22.

In the previous pilot study, we found that 16 T2DM‐related SNPs are of high level of variability and significant ethnic‐specific differences in Uyghur population compared with the other ethnic groups 18. This study aimed to further explore the possible association between 13 SNPs susceptibility loci and T2DM as well as the combined effects of these loci on the susceptibility of T2DM in a Uyghur population.

## Materials and methods

### Study participants

Between April 2012 and July 2013, we recruited a total of 265 Uyghur participants (130 T2DM patients and 135 healthy controls) from *Hetian* of *Xinjiang*, China, where the Uyghur population was less affected by the recent migration of Han Chinese. Both recruited cases and controls were not directly biologically related, and in addition they had no intermarriage history with other ethnic groups within the latest three generations. All subjects underwent routine health check‐ups at local *Minfeng Renmin* Hospital in *Hetian*. Diagnosis of T2DM was made by physicians according to 1999 World Health Organization (WHO) Criteria (fasting plasma glucose greater than or equal to 7.0 mmol/l and/or 2‐hrs plasma glucose greater than or equal to 11.1 mmol/l) 23. Biochemical [fasting blood glucose (FBG), serum total cholesterol (TC), triglycerides (TG), high‐density lipoprotein cholesterol (HDL), and low‐density lipoprotein cholesterol (LDL)] and anthropometric measurements [height, weight, body mass index (BMI)] were conducted as earlier described 18. Blood pressure was measured with a standard mercury sphygmomanometer in a sitting position after at least a 5 min. rest. Peripheral blood samples for analyses of biochemical indexes were collected in ethylenediaminetetraacetic acid anti‐coagulated tubes after an overnight fast. The concentration of FBG was determined by the glucose oxidase‐peroxidase method using commercial kits. Total cholesterol levels were analysed using cholesterol oxidase–peroxidase–amidopyrine method, TG by glycerol phosphate oxidase–peroxidase–amidopyrine method, HDL and LDL by enzymatic methods on a Hitachi 911 automated analyzer (Boehringer Mannheim, Mannheim, Germany).

All of the participants signed the informed consent before participation. This study was approved by the Ethical Committees of Xinjiang University, Urumqi, China and Capital Medical University, Beijing, China.

### Selection of the candidate SNPs

Thirteen SNPs [rs7754840 (*CDKAL1*), rs13266634 (*SLC30A8*), rs4402960 (*IGF2BP2*), rs1501299 (*APM1*), rs2241766 (*APM1*), rs2237892 (*KCNQ1*), rs2237895 (*KCNQ1*), rs35767 (*IGF1*), rs3792267 (*CAPN10*), rs10483776 (*FUT8*), rs7159888 (*FUT8*), rs3760776 (*FUT6*), and rs7953249 (*HNF1α*)] were selected for the following reasons. Firstly, we analysed nine SNPs from seven genes that had a nominal to strong association with T2DM identified by genome‐wide association studies (GWAS) among populations of European or Asian ancestry, *i.e*., *APM1*,* IGF1*,* CDKAL1*,* IGF2BP2*,* SLC30A8*,* KCNQ1*, and *CAPN10* 24. *CAPN10* gene has been identified as the first genetic locus susceptible to T2DM by positional cloning in Finland 25. In addition, four SNPs [rs10483776 (*FUT8*), rs7159888 (*FUT8*), rs3760776 (*FUT6*), rs7953249 (*HNF1α*)] were selected based on their associations with plasma fucosylation status 26. Although more and more susceptibility loci for T2DM are being identified, such glycosylation linked foundational loci have not yet been examined up to date. Minor allele frequencies (MAF) of all selected SNPs are more than 0.05 in both HapMap CEU data and HapMap CHB data (http://hapmap.ncbi.nlm.nih.gov/).

### SNPs genotyping

Genomic DNA was isolated from 200 μl venous blood samples using QIAamp DNA Blood Mini Kit according to the manual instructions (Qiagen Inc., Hilden, Germany). The concentration and purity of the isolated DNA were measured using the Thermo Scientific NanoDrop 2000 spectrophotometer according to the manufacturer. The DNA samples were genotyped by using Sequenom MassARRAY iPLEX Platform (Sequenom Inc., San Diego, CA, USA) 18. The PCR was executed in a 5 μl volume containing 1.8 μl deionized H_2_O, 0.5 μl 10× PCR buffer, 0.4 μl 25 mM MgCl_2_, 0.1 μl of 25 mM dNTP mix, 1 μl 0.5 μM primer mix, 0.2 μl (1 U) of PCR Hotstar enzyme, and 1 μl sample DNA. The PCR was performed in an ABI GeneAmp^®^ PCR system 9700 thermal cycler (384 dual) with the following conditions: denaturation at 95°C for 2 min. followed by 45 cyclers of 95°C for 30 sec., 56°C for 30 sec., 72°C for 1 min., followed by 72°C for 5 min. After each sample amplification, the PCR products were passed through a cocktail of 1.53 μl H_2_O, 0.17 μl of 10× shrimp alkaline phosphatase (SAP) buffer, 0.3 μl (0.5 U) of SAP (Sequenom Inc.). This was incubated 40 min. at 37°C, followed by 5 min. at 85°C and afterwards at 4°C for short‐term storage of the reaction. The single base extension reaction contained 0.619 μl H_2_O, 0.2 μl of 10× iPLEX^®^ Gold buffer, 0.2 μl of iPLEX^®^ Termination mix, 0.94 μl of primer mix (Sequenom Inc.), 0.041 μl of iPLEX^®^ enzyme, and 7 μl SAP treated PCR products. The single base extension reaction was performed in an ABI GeneAmp^®^ PCR system 9700 thermal cycler (384 dual) with the following conditions: denaturation at 94°C for 30 sec. followed by 40 cyclers of 94°C for 5 sec., 52°C for 5 sec., 80°C for 5 sec., 52°C for 5 sec., 80°C for 5 sec., 52°C for 5 sec., 80°C for 5 sec., 52°C for 5 sec., 80°C for 5 sec., 52°C for 5 sec., 80°C for 5 sec., followed by 72°C for 3 min. A total of 16 μl molecular grade water and 6 mg clean resin (Sequenom Inc.) was added to each sample. Sample plates were rotated on a rotator for approximately 35 min. and centrifuged at 3200 g for 3 min. The single base extension reaction products were spotted onto a SpectroCHIP array (Sequenom Inc.) using MassARRAY^®^ nanodispenser RS1000 (Sequenom Inc.). Then matrix‐assisted laser desorption/ionization time‐of‐flight mass spectrometry was performed and results were visualized on the MassARRAY^®^ compact system (Sequenom Inc.) using autorun settings. The three point calibration generated was at the m/z = 5044.4, 8486.6, 9977.0 D for the spectral analysis of all SNPs. The genotype call rate for each SNP exceeded 95% for both T2DM patients and healthy controls.

### Statistical analysis

All continuous variables were expressed as the mean ± S.D. Normality of distribution of all continuous variables was verified using the Kolmogorov–Smirnov tests. Continuous variables between T2DM and control groups were compared by Student's *t*‐test or rank sum test according to the results of Normality tests. Hardy–Weinberg equilibrium (HWE) for genotype frequencies was tested in both groups using chi‐squared test. *P* ≥ 0.05 was considered to obey the HWE. We considered the dominant and recessive genetic models in which the genotype was assigned as 0 or 1, depending on whether subjects carried the minor allele (genotype 1 for minor allele carrier, 0 for not, under dominant model; genotype 1 for homozygous for minor allele, 0 for not, under recessive model) 27. Allelic frequencies in T2DM patients and controls were compared by chi‐squared test, and logistic regression analyses were applied to evaluate differences in genotype distributions. Odds ratios (ORs) were calculated after adjustment for age, gender and BMI. In addition, to evaluate the combined effects of the SNPs, the cumulative risk allelic scores were calculated, based on the results of association analysis of candidate SNPs for T2DM. In such cases, we considered an additive genetic model for each SNP, and assigned a score of 0, 1, or 2 to the genotypes for the 13 loci, depending on whether subjects carried the wild‐type allele or were heterozygous or homozygous for the risk allele 18. The count method assumed that each risk allele contributes equally and independently to the risk for T2DM. The scores were modelled as a continuous variable and categorized into quartiles. The effects of cumulative risk allelic scores, obesity/overweight, and the interaction between the cumulative number of risk alleles and obesity/overweight on the risk of T2DM were tested using multivariate logistic regression. A two‐sided *P* < 0.05 was considered statistically significant.

The statistical analysis was conducted using SPSS for Windows, version 17.0 (SPSS Inc., Chicago, IL, USA). Power calculations were performed under current sample size and MAF observed in this study using Quanto software version 1.2.4 (http://hydra.usc.edu/gxe).

## Results

### Sample characteristics

The demographic and clinical profiles of 265 Uyghur participants (130 T2DM patients *versus* 135 healthy controls) are presented in Table 1. The T2DM patients had significantly higher levels of BMI, SBP, DBP, TC, TG, LDL and FBG compared to the controls (*P* < 0.05). Significant difference was found neither in gender, nor in HDL between the T2DM patients and healthy controls.

**Table 1:** Demographic characteristics and biochemical measures of the study participants

	T2DM (*n* = 130)	Control (*n* = 135)	*P*‐value
Age (years)	58.60 ± 11.15	56.36 ± 16.81	0.200
Gender (male)	59 (45.4%)	52 (38.5%)	0.258
BMI (kg/m^2^)	25.49 ± 3.51	24.08 ± 3.61	**0.001**
SBP (mmHg)	134.19 ± 18.54	120.41 ± 13.13	**<0.001**
DBP (mmHg)	90.50 ± 13.42	80.42 ± 9.31	**<0.001**
TC (mmol/l)	4.99 ± 0.99	4.01 ± 1.16	**<0.001**
TG (mmol/l)	1.88 ± 0.87	1.53 ± 0.70	**0.001**
HDL (mmol/l)	1.24 ± 0.30	1.24 ± 0.27	0.979
LDL (mmol/l)	2.83 ± 0.90	2.38 ± 0.76	**<0.001**
FBG (mmol/l)	8.78 ± 2.42	5.33 ± 0.66	**<0.001**

Data are shown as mean ± S.D. The *P*‐values with statistical significance are indicated in bold numbers. SBP: systolic blood pressure; DBP: diastolic blood pressure; TC: Total cholesterol; TG: Triglycerides; BMI: body mass index; FBG: fasting blood glucose.

### Association analysis of candidate SNPs for T2DM

**Figure 1:**
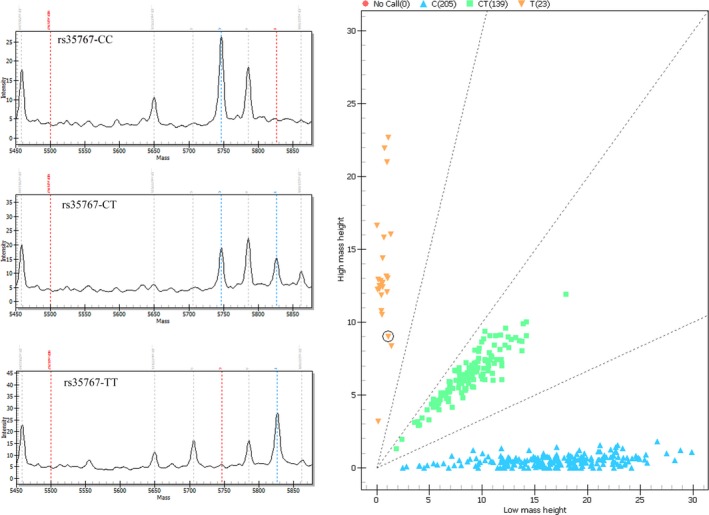
Matrix‐assisted laser desorption/ionization time‐of‐flight single‐nucleotide polymorphism mass spectrum and call cluster plot.

A representative mass spectrum for SNP genotyping was shown in Figure 1, and the assay information (*i.e*. homogeneous *versus* heterozygous SNPs) was initially assessed by call cluster plot analysis. The distributions of allelic and genotype frequencies of these 13 SNPs among the T2DM patients and controls, and the results from the logistic regression analysis are given in Tables 2 and 3. The distributions of allelic frequencies of the 11 SNPs conformed to HWE in both cases and controls (*P* > 0.05), except those of the rs2241766 and rs7159888 (*P* < 0.05) in the T2DM patients. The MAF of these SNPs were ranging from 0.11 to 0.52.

**Table 2:** Individual effects of the 13 candidate SNPs for T2DM in the Uyghur participants

Locus	dbSNP	A/a	T2DM		Control		Chi‐square test	Logistic regression analysis (adjusted for age, gender and BMI)	
			*F*_minor_	*P*_HWE_	*F*_minor_	*P*_HWE_	*P*‐value	OR (95% CI)	*P*‐value
*CAPN10*	rs3792267	G^a^/A	0.20	0.90	0.11	0.69	**0.004**	2.24 (1.35–3.71)	**0.002**
*APM1*	rs1501299	G^a^/T	0.19	0.84	0.27	0.44	**0.028**	0.59 (0.39–0.91)	**0.017**
*FUT6*	rs3760776	C^a^/T	0.11	0.82	0.17	0.51	0.071	0.57 (0.34–0.95)	**0.031**
*IGF1*	rs35767	C/T^a^	0.28	0.91	0.20	0.39	**0.029**	1.48 (0.98–2.23)	0.062
*KCNQ1*	rs2237892	C^a^^/^T	0.18	0.51	0.17	0.63	0.753	1.05 (0.66–1.67)	0.841
*KCNQ1*	rs2237895	A/C^a^	0.36	0.60	0.39	0.19	0.458	0.85 (0.59–1.22)	0.381
*APM 1*	rs2241766	T/G^a^	0.20	0.004	0.22	0.51	0.606	0.94 (0.61–1.44)	0.780
*IGF2BP2*	rs4402960	G/T^a^	0.31	0.17	0.25	0.53	0.140	1.26 (0.85–1.87)	0.243
*SLC30A8*	rs13266634	C^a^/T	0.28	0.74	0.26	0.77	0.645	1.11 (0.75–1.64)	0.617
*CDKAL1*	rs7754840	G/C^a^	0.38	0.64	0.32	0.19	0.122	1.29 (0.89–1.88)	0.171
*FUT8*	rs10483776	A^a^/G	0.14	0.78	0.15	0.51	0.681	0.86 (0.52–1.42)	0.548
*FUT8*	rs7159888	A^a^/G	0.35	0.02	0.40	0.89	0.235	0.81 (0.56–1.16)	0.246
*HNF1α*	rs7953249	G/A^a^	0.52	0.91	0.47	0.31	0.191	1.26 (0.88–1.79)	0.203

a Risk allele. A/a: major allele/minor allele. The *P*‐values with statistical significance are indicated in bold numbers. *P*_HWE_: *P*‐value of Hardy–Weinberg equilibrium test; *F*_minor_: minor allele frequency; *CAPN10*: calpain 10; *APM1*: aminopeptidase M1; *FUT6*: fucosyltransferase 6; *IGF1*: insulin‐like growth factor 1; *KCNQ1*: potassium voltage‐gated channel, subfamily Q, member 1; *IGF2BP2*: insulin‐like growth factor 2 mRNA binding protein 2; *SLC30A8*: solute carrier family 30 member 8; *CDKAL1*: cyclin‐dependent kinase 5 regulatory subunit associated protein 1–like 1; *FUT8*: fucosyltransferase 8; *HNF1α*: hepatocyte nuclear factor 1, alpha.

**Table 3:** Frequencies of the genotypes of rs3792267 (*CAPN10*), rs1501299 (*APM1*) and rs3760776 (*FUT6*) in T2DM patients and controls

SNP	Genotype	T2DM no. (%)	Control no. (%)	*P* *	Logistic regression analysis (adjusted for age, gender and BMI)†			
					Dominant		Recessive	
					*P*	OR (95% CI)	*P*	OR (95% CI)
rs3792267 (*CAPN10*)	GG	82 (63.1%)	108 (80%)	**0.006**	**0.002**	2.51 (1.42–4.46)	0.204	2.55 (0.60–10.82)
	GA	42 (32.3%)	25 (18.5%)					
	AA	5 (3.8%)	2 (1.5%)					
rs1501299 (*APM1*)	GG	85 (65.4)	69 (51.1)	**0.034**	**0.009**	0.51 (0.31–0.85)	0.732	0.91 (0.54–1.53)
	GT	39 (30)	56 (41.5)					
	TT	5 (3.8)	8 (5.9)					
rs3760776 (*FUT6*)	CC	102 (78.5)	94 (69.6)	0.113	**0.049**	0.56 (0.31–0.99)	0.211	0.33 (0.06–1.86)
	CT	26 (20)	36 (26.7)					
	TT	2 (1.5)	5 (3.7)					

*The *P*‐values for comparison of statistically difference among the three genotypes for certain SNP between T2DM and control subjects. ^†^The logistic regression model was used to obtain the odds ratios of the minor allele with the major allele as reference group. The *P*‐values with statistical significance are indicated in bold numbers.

Allelic frequencies of three SNPs [rs3792267 (*CAPN10*), rs1501299 (*APM1*), and rs3760776 (*FUT6*)] were significantly different between the T2DM and controls (*P* < 0.05). For rs3792267 (*CAPN10*), frequency of the A allele was significantly higher in T2DM patients than that in control group (0.20 *versus* 0.11, *P* = 0.004). For rs1501299 (*APM1*), frequency of the T allele was significantly lower in T2DM patients than that in control group (0.19 *versus* 0.27, *P* = 0.028). For rs3760776 (*FUT6*), frequency of the T allele was lower in T2DM patients than that in controls, although did not show the statistical significance (0.11 *versus* 0.17, *P* = 0.071). Multiple logistic regression analysis (adjusted for age, gender and BMI) identified that participants with the A allele for rs3792267 (*CAPN10*) had a 2.24‐fold [OR (95% CI): 1.35–3.71, *P* = 0.002] risk of T2DM compared with the G allele. The T alleles at rs1501299 (*APM1*) and rs3760776 (*FUT6*) were also found to be significantly associated with T2DM in logistic regression analysis [OR (95% CI): 0.59 (0.39–0.91), *P* = 0.017 *versus* OR (95% CI): 0.57 (0.34–0.95), *P* = 0.031] (Table 2). For rs35767 (*IGF1*), frequency of the T allele was significantly higher in T2DM patients than that in controls (0.28 *versus* 0.20, *P* = 0.029), although did not show the statistical significance after age, gender and BMI adjusted [OR (95% CI): 1.48(10.98–2.23, *P* = 0.062)] (Table 2). Contrary to SNP rs3792267/GG, SNPs rs1501299/GG and rs3760776/CC were more frequent in the T2DM group compared to the controls (63.1% *versus* 80%; 65.4% *versus* 51.1%; 78.5% *versus* 69.6%, respectively; Table 3).

To evaluate the combined effects of these 13 associated variants, we calculated the cumulative risk allelic scores of these 13 risk alleles that each participant had using a simple count approach. The average of cumulative risk allelic scores of T2DM patients (15.15 ± 2.19) was significantly higher than that in controls (14.16 ± 2.22) (*P* = 0.001, *t*‐test). Multivariable logistic regression analyses also indicated that risk allelic scores were important factors of T2DM in Uyhgur participants (*P* = 0.001).

To further examine the association between obesity/overweight and T2DM across four categories of risk allelic scores, *i.e*., quartile 1 (Q1) (risk allelic scores less than or equal to a value of 14), quartile2 (Q2) (risk allelic scores equal to a value of 15 or 16), quartile 3 (Q3) (risk allelic scores equal to a value of 17 or 18) and quartile 4 (Q4) (risk allelic scores greater than or equal to a value of 19), we stratified the subjects into two groups: the obese/overweight group (BMI ≥24 kg/m^2^) and the non‐obese group (BMI <24 kg/m^2^). Obesity/overweight was a strong predictor of T2DM in the Uyghur participants (*P* = 0.004). However, obesity/overweight was not shown as a significant risk factor for T2DM (*P* > 0.05) for the subjects in the Q3 and Q4 groups (Table 4).

**Table 4:** Effect of obesity/overweight on T2DM according to quartiles of risk allelic scores

Quartiles of risk alleles	T2DM (*n* = 130)	Control (*n* = 135)	OR^a^	95% CI	*P*
All subject					
Obese	82	62	2.075	1.263–3.407	**0.004**
Non‐obese	48	73			
Q1 (≤14)					
Obese	28	33	2.158	1.002–4.647	**0.049**
Non‐obese	19	43			
Q2 (15, 16)					
Obese	32	18	2.431	1.022–5.782	**0.044**
Non‐obese	16	22			
Q3 (17, 18)					
Obese	18	10	0.667	0.150–2.973	0.595
Non‐obese	9	4			
Q4 (≥19)					
Obese	4	1	4.978	0.306–81.083	0.26
Non‐obese	4	4			

a ORs and *P*‐values on T2DM for obese/overweight and non‐obese subjects were adjusted for age. The *P*‐values with statistical significance are indicated in bold numbers.

## Discussion

Genome‐wide association studies have successfully identified over 70 loci associated with the risk of T2DM in multiple populations, especially in populations of European ancestry 28. However, the risk attributable to an individual variant to date is modest and does not yet provide convincing evidence for clinical utility. Genetic studies have revealed that different populations have different genetic structures because of their complex demographic histories 29. In addition, the heterogeneity of drug responses further illustrates the genetic variants vary substantially among different Chinese ethnic groups 22. Therefore, current available data are not likely to be applicable to all populations. Uyghur is a classically well‐defined isolated population, practicing endogamy resident in a relatively homogeneous environment and having large sibships. Therefore, it would be an ideal population for the study of genetic susceptibility.

### Study findings

In this population‐based case–control study, we extended the support for T2DM candidate loci identified by GWAS 24 and revealed that the cumulative risk allelic scores that aggregate information from multiple genetic variants are significant risk factors in the Uyghur population. Among the 13 T2DM genetic susceptibility loci screened, we found significant association with T2DM for three of them, *i.e*., rs3792267 (*CAPN10*), rs1501299 (*APM1*) and rs3760776 (*FUT6*).

*CAPN10* gene, identified as the first susceptibility gene for T2DM by positional cloning 25, has been associated the increased risk of T2DM in different populations 30, 31, 32. It has been well documented that the abnormal expression of *CAPN10* in pancreatic islets, muscle and liver is related to insulin secretion and action, and thereby is considered to be an important novel pathway involved in glucose metabolism 25. Single‐nucleotide polymorphism rs3792267polymorphism in *CAPN10* has been examined on the effect of regulating insulin sensitivity and *CAPN10* mRNA levels 33. However, divergent results were reported and gave a heterogeneous picture owing to racial or regional differences 31, 32, 34, 35. Our study revealed that the A allele of rs3792267 of *CAPN10* was significantly associated with T2DM in Uyghur participants (*P* = 0.002, adjusted for age, gender and BMI) (Table 2).

A significant association was detected between the A allele of rs3792267 (*CAPN10*) and T2DM susceptibility in the dominant model (*P* = 0.002; Table 3). Kommoju *et al*. showed that the increased A allelic frequency of rs3792267 in the T2DM patients than that of controls in a Indian population of Hyderabad 36, conferring the risk for developing T2DM. Another study conducted on the Kurdish ethnic group of Iran population also supported the association of A‐allele of rs3792267 with T2DM 10, which was consistent with the results of this study for Uyghur participants. Furthermore, Li *et al*. also reported association of the A allele with high risk of T2DM in Uyghur population 6. Yet, some studies showed two opposite trends of statistical significance of the G‐ and A‐allele towards T2DM in certain population 34. This discrepancy suggested that the association should be population‐specific due to the differential allelic frequencies across different human ethnic groups. The distribution of the ancestral G allele at rs3792267 in controls reported in previous studies varied from 0.68 to 0.97 in different populations such as European 25, 37, 38, Arabic 39, African‐American 40, South Indian 41, Japanese 42 and Chinese 43. The frequency of the G allele for rs3792267 in Uyghur controls (0.893) was reported to be similar level with African‐Americans, Tunisian Arab and South Indian, but higher than that in Caucasian populations from Britain and Ireland, and lower than that in Japanese. Uyghur, as one of typical Muslim minorities 44, has high levels of consanguineous and/or endogamous marriage which might increase the likelihood of presence of pathogenic mutations at a higher homozygous level, with mean inbreeding coefficient of 0.0033–0.0065 45. Therefore, the accumulation of various mutations due to endogamy seems to affect the distribution of allele frequency across different population groups 18, 46, 47.

Among the other loci examined in this study, SNP rs1501299 in *APM1* also showed the significant association with T2DM in Uyghur participants (*P* = 0.017, adjusted for age, gender and BMI, Table 2). Single‐nucleotide polymorphism rs1501299 locates in intron‐2 within the *APM1* gene, which plays a pivotal role in regulating insulin sensitivity 48. In this case–control study, the frequency of GG genotype of rs1501299 was noted significantly higher in T2DM patients as compared with that of controls, suggesting that the G allele may confer increased risk for T2DM. Indeed, a significant association was detected between the G allele of rs1501299 and T2DM susceptibility in the dominant model (Table 3). Tu *et al*. reported that the rs1501299 polymorphism was associated with increased risk for T2DM, especially in Chinese Han population 49. The direction and magnitude of our result were consistent with these previous reports 50, 51.

*FUT6* gene, as a member of fucosyltransferase (FUT) family, is involved in catalysing the inverting reaction, in which a fucose residue is transferred from guanosine‐diphosphate fucose (GDP‐Fuc) to molecules such as *N*‐glycans 52. Single‐nucleotide polymorphism rs3760776, in the promoter region of *FUT6* 53, was also reported to have association with plasma levels of fucosylated glycans such as DG7 (*P* = 3.42 × 10^−12^) and DG9 (*P* = 3.51 × 10^−17^) 26. In mammals, fucose‐containing glycans have been shown to be associated with the host‐microbe interactions, transfusion reaction and selectin‐mediated leucocyte‐endothelial adhesion 54, 55. In our case–control study, the frequency for the T allele in rs3760776 was noted significantly lower in diabetic patients as compared with that of controls (*P* = 0.031). In logistic regression analysis, marginal association for SNP rs3760776 was detected in dominant model (OR = 0.56, 95% CI: 0.31–0.99, *P* = 0.049) (Table 3). Single‐nucleotide polymorphism rs3760776 may affect the activity of *FUT6* enzyme, and thus may alter individual's capacity to fucose conversion and in turn modify the risk in the development of T2DM. The study on the glycosylation profiling of this same cohort of Uyghur T2DM patients is currently under way in our laboratory to understand the molecular mechanism underlying the statistically significant associations observed in this study.

Although significantly associated with T2DM, the risk attributable to an individual variant is modest, which limits the clinical utility. However, taken collectively, a combination of information from multiple genetic variants may contribute substantially to the disease risk and will be useful in characterizing population at high risk for T2DM. We have shown that the cumulative risk allelic scores based on the 13 susceptible loci for T2DM are significant risk factors in the Uyghur population samples (*P* = 0.001), consistent with several previous studies in Asian populations 18, 56, 57. In addition, our data suggested that obesity/overweight is a strong predictor of T2DM (*P* = 0.004), given obesity is associated with insulin resistance 58. However, obesity/overweight was not shown as a significant risk factor of T2DM for the patients with the combined risk allelic scores greater than or equal to the value of 17, perhaps due to the small sample size in the subgroups, such as Q3 and Q4, with insufficient statistical power. This result was comparable with the study among a Japanese population 59, in which most T2DM patients were characterized by a low BMI and shared the same pattern of genetic profiling on the T2DM susceptible loci.

### Study limitations

Our study has limitations. A relatively small sample size: the blood sampling of minority groups was a very hard field‐practice in the remote areas of *Xinjiang* 18, 22. Thereby, the combined data set provided statistical power of less than 80% (40.9–64.7%) to detect SNPs with risk ratios greater than 1.5, given the significance level of 0.05. Thus, case–control studies with large samples and multiple comparisons are required to confirm the effect of those SNPs on the T2DM risk based on this current observation. Moreover, GWAS identify SNPs and other DNA variants associated with a disease, but cannot on their own specify which genes are causal 60, 61. Thereby, function studies in carefully selected study participants and animal model are also needed to specify the molecular mechanism underlying the statistical association observed.

## Conclusion

The SNP rs3792267 in *CAPN10*, rs1501299 in *APM1*, and rs3760776 in *FUT6* might serve as potential susceptibility loci for T2DM in Uyghurs. The cumulative risk allelic scores of multiple loci with modest individual effects are also significant risk predictors/factors in Uyghurs for T2DM, particularly among non‐obese individuals. This is the first investigation having observed/found genetic variations on genetic loci functionally linked with glycosylation associated with the risk of T2DM in a Uyghur population.

## Conflict of interest

None.

## Author contribution

WW and MSS designed the study, wrote and revised the manuscript. FFZ performed the analysis and interpretation of data, and draft the manuscript. MD contributed to acquisition of data (population studies) and reviewed the manuscript. YXW contributed to acquisition of data (genetics) and reviewed the manuscript. HHF, HW, QG, HD, SQG, XYY, JZ and LJW provided technical support for the analysis and interpretation of data, and critical revision of the manuscript. All authors read and approved the final manuscript.
