# Correction to “The Uyghur Population and Genetic Susceptibility to Type 2 Diabetes: Potential Role for Variants in CAPN10, APM1 and FUT6 Genes”

**DOI:** 10.1111/jcmm.70638

**Published:** 2025-05-30

Zhao F, Mamatyusupu D, Wang Y, Fang H, Wang H, Gao Q, Dong H, Ge S, Yu X, Zhang J, Wu L, Song M, Wang W. The Uyghur Population and Genetic Susceptibility to Type 2 Diabetes: Potential Role for Variants in CAPN10, APM1 and FUT6 genes. *J Cell Mol Med*. 2016;20(11):2138‐2147.

In the Acknowledgements section, the Australian National Health and Medical Research Council (NHMRC‐APP1112767) and the Edith Cowan University Strategic Research Fund (SRF‐2015) should be removed.

The Acknowledgement statement reads:

This study was supported by research grants from the National Natural Science Foundation of China (81573215, 81273170, 31460285 and 81370083), National 12th Five‐Year Major Projects of China (2012BAI37B03), Australian National Health and Medical Research Council and National Natural Science Foundation of China (NHMRC‐APP1112767‐NSFC 81561128020), Edith Cowan University Strategic Research Fund (SRF‐2015), Natural Science Foundation of Xinjiang Uyghur Autonomous Region (2013211A016), and Natural Science Foundation of Capital Medical University, Beijing, China (2014ZR16). Manshu Song was supported by the Importation and Development of High‐Calibre Talents Project of Beijing Municipal Institutions (CIT&TCD201404185). The authors thank the Uyghur volunteers and community leaders for their supports and participation. We appreciate the English editing by Eric Adua, School of Medical Sciences and Health, Edith Cowan University, Australia.

The Acknowledgement statement should read:

This study was supported by research grants from the National Natural Science Foundation of China (81573215, 81273170, 31460285, 81370083 and 81561128020), National 12th Five‐Year Major Projects of China (2012BAI37B03), Natural Science Foundation of Xinjiang Uyghur Autonomous Region (2013211A016) and Natural Science Foundation of Capital Medical University, Beijing, China (2014ZR16). Manshu Song was supported by the Importation and Development of High‐Calibre Talents Project of Beijing Municipal Institutions (CIT&TCD201404185). The authors thank the Uyghur volunteers and community leaders for their supports and participation. We appreciate the English editing by Eric Adua, School of Medical Sciences and Health, Edith Cowan University, Australia.

We apologize for this error.

